# Creation and validation of a roadside rescue skills scale for training pre-hospital medical teams: the RoadRes-Q scale

**DOI:** 10.1186/s13049-025-01370-x

**Published:** 2025-04-03

**Authors:** Killien Lavabre, Nicolas Marjanovic, Denis Oriot, Mathilde Chenu, Adrien Gransagne, Michel Gentilleau, Anthony Moreau, Paul Contal, Olivier Mimoz, Bertrand Drugeon, Road Rescue Study Group, Jean Gabriel Clouet, Tom Van Esbroeck, Sébastien Guay, Ruediger Knoll, Julien Manesse, Frans Van Maurik, Bart Noyens, Raphael Mouth, Cédric Rigollet

**Affiliations:** 1 Service des urgences adultes et SAS 86 / SMUR, CHU de Poitiers, 2 rue de la Milétrie, 86021 Poitiers Cedex, France; 2 INSERM CIC-1402, IS- ALIVE, Université de Poitiers, Poitiers, France; 3 ABS Lab, Faculté de Médecine et de Pharmacie, Université de Poitiers, Poitiers, France; 4 Service des Urgences et SAMU, Hôpital Saint Louis, La Rochelle, France; 5 Service Départemental d’Incendie et de Secours 86, Chasseneuil du Poitou, France; 6 PHAR2 - INSERM U1070, Université de Poitiers, Poitiers, France; 7 Service de protection et sauvetage Lausanne, Lausanne, Switzerland; 8 Center of expertise in Belgium, directorate general for civil security, Brandweerzone Centrum, Ghent, Belgium; 9 Institut de protection contre les incendies du Québec, Québec, Canada; 10 Weber rescue systems, Güglingen, Germany; 11 Service Départemental d’Incendie et de Secours 17, La Rochelle, France; 12 RIFRA Instructions, Nieuwegein, Netherlands; 13 Belgian Federal Working Group on Technical Rescue, Brussels, Belgium; 14 Falck, Montigny le Bretonneux, France; 15 Service Départemental d’Incendie et de Secours 51, Fagnieres, France

## Abstract

**Background:**

Road traffic injuries are the leading cause of death among young people worldwide. While advances in vehicle safety have reduced some of the risks, the speed and quality of pre-hospital care are critical to prevent fatalities. In France, patients are cared for by medical teams and firefighters who must work together as closely as possible to ensure the best possible survival rate. However, there is a lack of standardised scales to assess the performance of these multidisciplinary teams. This study aimed to create and validate a roadside rescue skills assessment scale, the RoadRes-Q scale, for healthcare teams.

**Methods:**

We used a two-round Delphi method to develop the RoadRes-Q scale. A panel of 9 international roadside rescue experts, including 7 firefighters and 2 engineers in road rescue equipment, agreed to participate. The scale covers five key areas: healthcare provider protection, site securing, vehicle securing, first aid delivery, and patient extrication. The final version was tested during two one-day simulation-based training sessions, each involving 22 participants: 6 healthcare staff, 14 firefighters, and 2 simulated victims. Assessors completed the scale during and after each scenario, focusing on internal consistency and inter-observer reliability.

**Results:**

The RoadRes-Q scale consists of 60 items. Internal consistency was excellent (Cronbach’s alpha of 0.86), indicating that items were non-redundant and consistently measured the required competencies. However, inter-observer reliability was low (intra-class correlation coefficient of 0.48), suggesting variability between assessors. Satisfaction among participants to the simulation-based training courses was high, and their knowledge increased.

**Conclusions:**

The RoadRes-Q scale proved to be a valid and reliable scale for evaluating both technical and non-technical skills. While internal consistency was strong, improvements are needed in inter-observer reliability. Structured training for assessors and video-based assessments could enhance reproducibility. The RoadRes-Q scale has the potential for assessing the quality and safety of care provided by healthcare teams in roadside rescue situations.

**Registration:**

As the study did not involve interventional research or patient participation, ethics committee approval was not required, but it received approval from the scientific referents of the Faculty of Medicine of Poitiers, and participants provided informed consent for using their anonymised data.

## Introduction

Each year, approximately 1.2 million people lose their lives in road traffic accidents, making it the leading cause of death among children and young adults aged 5–29. In addition, between 20 and 50 million people sustain non-fatal injuries, many of which result in long-term disabilities. These incidents also lead to significant economic burdens, affecting individuals, their families, and nation as a whole. The costs stem from medical treatment and the loss of productivity, both from those killed or disabled and from family members who must take time off work or school to provide care. Road traffic accidents account for 3% of the gross domestic product in most countries, reflecting both direct costs, such as medical expenses and emergency response; and indirect costs, including loss of productivity and long-term disabilities [1].

Despite improvements in vehicle safety and road infrastructures, the speed and quality of pre-hospital care remain critical, particularly in preventing fatalities. About 50% of deaths occur within minutes of the accident, either at the scene or in route to the hospital; 15% of fatalities occur between one- and four-hours post-collision, and the remaining 35% occur more than four hours after the event [2]. This highlights the urgent need for rapid and efficient pre-hospital interventions to mitigate the consequences of trauma.

In France, emergency medical call centres (SAMU—one per county) and their mobile emergency and resuscitation units (SMUR) play a central role in treating the most critical patients outside the hospital. Established since 1956 by the Ministery of Health, each SMUR team traditionally includes an emergency physician, a nurse, and an ambulance driver [3]. The French “stay and play” model contrasts sharply with the “scoop and run” approach used by Anglo-Saxon paramedics, where the priority is to transport the patient quickly to the nearest hospital with minimal on-site care [4, 5]. In contrast, the French model emphasises comprehensive care at the scene, utilising advanced technical equipment to stabilise patients before transport.

In parallel, French firefighters are highly versatile, tasked with a range of responsibilities that include firefighting, environmental risk management and emergency personal assistance, the latter being their most frequent mission [6]. Firefighters are often the first responders at the scene of road accidents, and specialised technical teams are deployed for complex rescues, such as roadside extrications. These operations require specialised vehicles, equipment, and techniques to safely remove patients trapped in vehicles, underscoring the technical complexity and coordination required during such emergencies. Given the crucial roles of both emergency medical teams and firefighters in these situations, the need for a shared culture and standardised training is essential. A common framework would ensure that both medical and technical teams operate seamlessly together, optimising patient outcome. The success of such high-stakes interventions depends on clear communication, leadership, and the ability to work under pressure [7]. Poor coordination or communication breakdowns can delay critical care, leading to worsen patient outcome [8]. This makes it essential to not only provide thorough training, but also develop reliable ways to evaluate the performance of these multidisciplinary teams working together.

Training methods for emergency responders have traditionally focused on theoretical knowledge and practical skills. However, in recent years, simulation-based training has emerged as a key educational pathway, offering realistic, controlled environments where teams can practice handling real-world scenarios without risk to patients [9, 10]. Despite its many advantages, simulation-based training for medical teams in roadside rescue remains rare. Furthermore, the absence of a standardised assessment scale means that there is no reliable way to measure the effectiveness of this training. Developing such a scale is a great challenge, as it requires capturing both technical and non-technical skills, such as communication and leadership, which are crucial for successful rescues. It will enable educators to not only assess but also enhance the skills of rescue teams, ultimately improving patient outcome.

To address this complexity, this study aims to develop a valid and reliable assessment scale specifically designed for medical teams participating in roadside rescue scenarios. It serves as a teaching scale during multidisciplinary simulation training sessions, allowing for objective evaluation of both technical and non-technical skills such as teamwork, leadership, and decision-making under pressure.

## Methods

### Study design and setting

This study was conducted in collaboration with the firefighters of the Vienne County (Service Départemental d'Incendie et de Secours [SDIS] 86), recognised as a national benchmark in France for their expertise in managing road traffic accidents. Their advanced techniques in accident scene safety, vehicle security, first aid, and patient extrication have significantly influenced the development of national guidelines [11–14]. Since 2018, these firefighters have been working closely with the emergency medical team from the Poitiers University Hospital to develop a shared set of guidelines for roadside rescue operations. The primary aim of this collaboration was to enhance the skills of all rescue actors—physicians, nurses, ambulance drivers, and firefighters—by creating a unified framework that focuses on the roadside rescue environment. Although the framework does not cover trauma-specific medical care, it provides clear protocols for managing the scene, securing vehicles, coordinating rescue efforts, and safely extricating patients.

The guidelines, co-authored by two emergency physicians and two firefighters with specialised expertise in roadside rescue, were built on both practical experience and a review of the relevant literature. The reference manual they produced covers the full sequence of roadside rescue operations, including general rescue procedures, vehicle-specific considerations, scene and vehicle security, patient management, and extrication techniques. Additionally, the manual also provides details on beaconing methods, patient discharge protocols, and specific equipment descriptions to standardise practices across all rescue teams. Although this manual is not yet publicly available, it is currently being updated and formatted for wider dissemination, with plans for a future printed publication in both French and English.

The present study employed a two-round Delphi method to develop a scale for assessing the competencies of medical teams in roadside rescue scenarios, called the RoadRes-Q scale. We recruited a panel of international experts. The Delphi method aimed to ensure a consensus-driven approach to the content of the assessment scale, which was then validated for internal consistency and inter-rater reliability during two one-day multi-disciplinary road rescue simulation-training sessions.

### Creation of the RoadRes-Q scale

#### Content drafting prior to the first Delphi round

The draft of the RoadRes-Q scale was grounded in a thorough review of existing guidelines and referential data on roadside rescue interventions. This process was structured using the Delphi method to ensure a systematic and consensus-driven approach [15, 16]. The initial framework of competencies and criteria was derived from established best practices in emergency and prehospital care, and organised into five main sections reflecting key operational domains:

- *Personal protection of responders*: This section covered the essential actions and precautions to ensure the safety of the medical team at the scene.
- *Site security*: This section explored criteria related to managing the safety of the overall scene, including controlling traffic flow, preventing additional hazards, and ensuring a secure working environment for all teams involved.
- *Securing vehicles*: This category focused on actions necessary to stabilise vehicles involved in the accident, preventing further movement or risk to both the responders and the victims.
- *First-aid intervention*: This section addressed the initial medical interventions, including patient triage, immediate care, inter-service communication, and ensuring continuity of care between different emergency services.
- *Patient extrication*: This final category outlined the steps for safely extricating patients from vehicles, coordinating their transport, and ensuring their protection during the process.

Each of these sections was subdivided into specific, measurable actions that aligned with real-world expectations during roadside interventions. A core working group, consisting of three senior emergency physicians and two experienced firefighters, reviewed and refined this initial list of competencies to ensure its practical relevance. This preliminary version of the RoadRes-Q scale served as the foundation for the first round of the Delphi method, ensuring that all items presented to the expert panel were organised logically and represented the critical aspects of roadside rescue interventions.

#### Expert panel selection

The experts were selected based on their expertise in prehospital emergency care and road traffic rescue operations, from among the authors of leading articles, trauma recommendations, from trauma networks, international judges at road rescue competitions and engineers from companies developing road rescue equipment. Each expert had at least five years of experience in their respective fields. Invitations were sent via email, and participants were informed of the purpose and structure of the Delphi method, which required their input over multiple rounds. Their consent to take part in the study was clearly expressed in their reply email.

#### Delphi process

After the initial version of the RoadRes-Q scale was developed, it was sent to the panel of experts for review. Each expert was asked to rate the relevance of each item on a 7-point Likert scale, ranging from 0 ("not at all relevant") to 6 ("very relevant"). Additionally, a free-text field was provided for experts to offer comments or suggestions for improving the items and/or adding some ones. The median score for each item was calculated after the first round of responses. Items with a median score below 4 were eliminated from the scale. For items with median scores between 4 and 5, revisions were made based on the feedback received to improve their clarity and relevance, with the aim of making these items "highly relevant" to the experts. Items that received a median score of 6 were retained without any modifications. Following this, a second version of the RoadRes-Q scale was created and distributed to the experts who participated in the first round for further assessment. During this second round, the median score for each item was recalculated, and items that received a median score between 5 and 6 were retained in the final version.

### Psychometric testing

#### Simulation training sessions

The RoadRes-Q scale was tested for two one-day sessions of multidisciplinary roadside rescue simulation training at the Vienne fire department training centre in Valdivienne, France. Prior to taking part, participants were given the road rescue guidelines and encouraged to consult them. Each one-day session involved 16 participants, comprising 14 firefighters and 2 simulated victims. Firefighters were split into four teams, with two first responders' ambulance teams and two roadside rescue teams. Each team was made up of 3 participants, which corresponds to the usual crew in real life. One rescue operations commander commanded both an ambulance team and a road rescue team. Six healthcare staffs were split into two SMUR teams, each consisting of an emergency physician, a nurse, and an ambulance driver. All participants were divided into two equal groups for each simulation scenario, i.e. one ambulance team, one roadside rescue team, one rescue operations commander, one SMUR team and one simulated victim. Six observers evaluated the medical team performance during the first one-day training session, and four the second one-day session. Each training day included two or three scenarios. Each scenario was carried out simultaneously and independently, allowing all participants to complete both scenarios. The day began with a reminder on key elements of the guidelines, including scene safety procedures, vehicle stabilisation techniques, initial patient assessment, coordinated team communication, and extrication protocols. This was followed by an introduction to specific rescue equipment, a hands-on airbag activation workshop, and the distribution of a cognitive aid sheet to assist with decision-making during the scenarios. The session continued with a pre-briefing led by a simulation training expert followed, introducing supervisors, outlining their roles, and reviewing the principles and benefits of simulation in improving patient care [17, 18]. The importance of learner engagement was emphasised, as were the limitations of simulation in fully replicating real-world situations. Participants were reminded that simulation offers a safe environment to practice and refine skills.

Each simulation session started with a briefing introducing the context, after which the teams began their operations. Scenarios ended when the simulated patient was safely extracted from the vehicle and stabilised for transport. If patient extraction took more than 40 min, a simulated cardiac arrest occurred, triggering an emergency extraction. Throughout the scenario, observers completed the RoadRes-Q scale to document the team's performance. Each simulation was followed by a 30-min debriefing session, using the after-action review (AAR) method, which is commonly employed by the US Army [19–22]. This structured debriefing process allowed participants to reflect on their actions, discuss areas for improvement, and consolidate their learning from the day.

#### RoadRes-Q scale application and validation

The RoadRes-Q scale was employed during each simulation session. Observers included three emergency physicians trained in simulation and debriefing, an academic educator and practitioner specialised in pediatrics care and simulation, a lieutenant from firefighter department with expertise in roadside rescue, a firefighter instructor with experience in first aid training, two ED nurses trained in simulation teaching, and two emergency medicine residents. Each observer received one hour's training during a preparatory meeting for the simulation days. These observers were responsible for completing the RoadRes-Q scale both during and immediately after each simulation scenario. They were all blinded to the evaluations of the others. The data collected through these assessments were used for the statistical analysis to study the validity and the reliability and consistency of the scale.

Each item was evaluated using a 3-point scale, where 0 represented “Not done”, 1 indicated “Done partially, unexpectedly, or inappropriately”, and 2 signified “Done correctly and on time”. For scenarios where an item could not be evaluated, a “Not Attributable” option was provided. This scoring system was based on the methodology of the Team Average Performance Assessment Scale (TAPAS) [23, 24]. Furthermore, key items in the RoadRes-Q scale highlighted specific actions that were expected when the medical team was the first to arrive at the scene of the accident, ensuring that the assessment reflected real-world expectations and operational priorities.

### Evaluation of training sessions

Demographic criteria of the medical teams involved in this study were analysed based on several key variables, including gender, age, years of experience in the ED, the approximate number of roadside rescue interventions performed, and prior roadside rescue training. Age was categorised into four groups (25–30, 30–35, 35–40, and > 40 years). The number of years of experience in the ED was grouped into three categories (1–5 years, 6–10 years, and > 10 years). Participants reported the approximate number of roadside rescue interventions they had performed, categorised into 1–9, 10–20, and > 20 interventions, allowing for an evaluation of practical experience within the team. Finally, participants were asked whether they had previously attended a roadside rescue training program, with responses classified as “yes, in Poitiers”, “yes, in another centre”, or “no”.

The quality of the training sessions was evaluated using the first two levels of the Kirkpatrick's pyramid: learners' satisfaction and gain in Knowledge, Skills and Attitudes (KSA). Learners' satisfaction was evaluated using a Likert scale from 1 to 5 (1 = ”strongly disagree” to 5 = ”strongly agree”), which measured self-assessment participants for the interest, the organisation of the training, its theoretical and practical relevance, and their overall satisfaction. Gain in knowledge was assessed through a 23-item multiple-choice questionnaire (MCQ), which was based on the developed roadside rescue guidelines. Medical teams completed the questionnaire online before (pre-test) and after (post-test) the training sessions.

Clinical skills were assessed using the TAPAS tool. TAPAS was designed to provide a performance assessment score for a multiprofessional team’s approach to a simulated life-threatening situation, covering both medical and traumatic emergencies in adults. Its objective is to assess both quality and response time, based on three categories: not performed (0/2), performed but incorrectly or delayed (1/2), and correctly performed on time (2/2). TAPAS yields a score out of 100.

Teamwork performance is assessed using the Clinical Team Scale (CTS), a tool designed for evaluating team effectiveness in clinical scenarios, especially in high-stakes simulations. CTS measures critical aspects of teamwork—such as communication, coordination, leadership, and situational awareness, through a 10-point rating scale for each item, where 1 signifies poor performance and 10 represents exemplary performance. This scoring method provides a nuanced evaluation of team dynamics, with a higher cumulative score indicating a well-coordinated, communicative, and supportive team. The item concerning family information could not be scored in the scenarios, so the maximum score was 130.

### Data analysis

#### Description of participants in simulation training sessions

Descriptive statistics such as number and percentages were calculated for each age group, measured using scale as described above.

#### Psychometric testing: validation of the RoadRes-Q scale

The validation of the RoadRes-Q scale focused on two key aspects: internal consistency and inter-observer reliability.

- Internal consistency:
  Internal consistency was analysed by calculating Cronbach's alpha coefficient (Cronbach LJ), which measures the degree to which items within the scale are correlated, thus assessing the reliability of the scale. According to Bujang et al. (2018), a Cronbach's alpha value greater than 0.5 is considered acceptable for exploratory research [25].
- Inter-observer reliability:
  Inter-observer reproducibility was assessed using different means:
  - Means and variances of each observer's score were calculated and compared with a T-Test and F-test respectively, which allowed for an assessment of whether the variability in scores was statistically significant. A p-value < 0.05 was considered indicative of statistical significance in all analyses.
  - Comparison of variances of the observers (F-Test)
  - Intra-Class Correlation coefficient (ICC). The ICC was selected to evaluate the agreement between different observers assessing the same medical team using the scale. ICC values were interpreted based on established guidelines: values below 0.5 indicate low reliability, values between 0.5 and 0.75 indicate moderate reliability, values between 0.75 and 0.90 indicate good reliability, and values above 0.90 indicate excellent reliability [26].
  - Linear regression analysis was performed to graphically represent the relationship between observer 1 and 2, as well as the calculation of R^2^, where a R^2^ above 0.5 indicates a strong reproducibility between observers.

#### Evaluation of training sessions

- *Learner satisfaction:* Descriptive analysis, including means, were performed on learners' satisfaction, measured using a Likert scale as described above.
- *Knowledge improvement:* The assessment of knowledge improvement was conducted through a 23-item MCQ administered both before and after the simulation training. A T-test was used to compare the pre-test and post-test scores of the medical teams, assessing whether there was a statistically significant increase in knowledge following the training. The difference between pre- and post-test scores was analysed to quantify the overall impact of the training. A p-value of < 0.05 was considered statistically significant, indicating meaningful knowledge acquisition between the pre- and post-test phases.
- Clinical skills and teamwork performances: Descriptive analysis, including means and standard deviation, were performed on TAPAS and CTS.

#### Software used for data analysis

All statistical analyses were performed using R software (Biocore, Boston, Massachusetts, USA), *stats*, *irr* and *lpSolve* packages [27–29], ensuring a rigorous approach to data analysis and reliability testing.

### Ethics

Since this study did not involve any interventional research or direct involvement of patients, approval from an ethics committee was not required. However, the study received approval from the scientific referents of the Faculty of Medicine, Poitiers, France. Additionally, all participants were informed about the study, and each provided consent for the use of anonymised data.

## Results

### RoadRes-Q scale development

A total of 22 experts from various countries were contacted in 2021, including professionals from France, Switzerland, Belgium, Canada, the Netherlands, Germany, Spain, Norway, Austria, and Italy. The panel consisted of 14 firefighters, six emergency physicians, and two engineers in road rescue equipment.

**Fig. 1:**
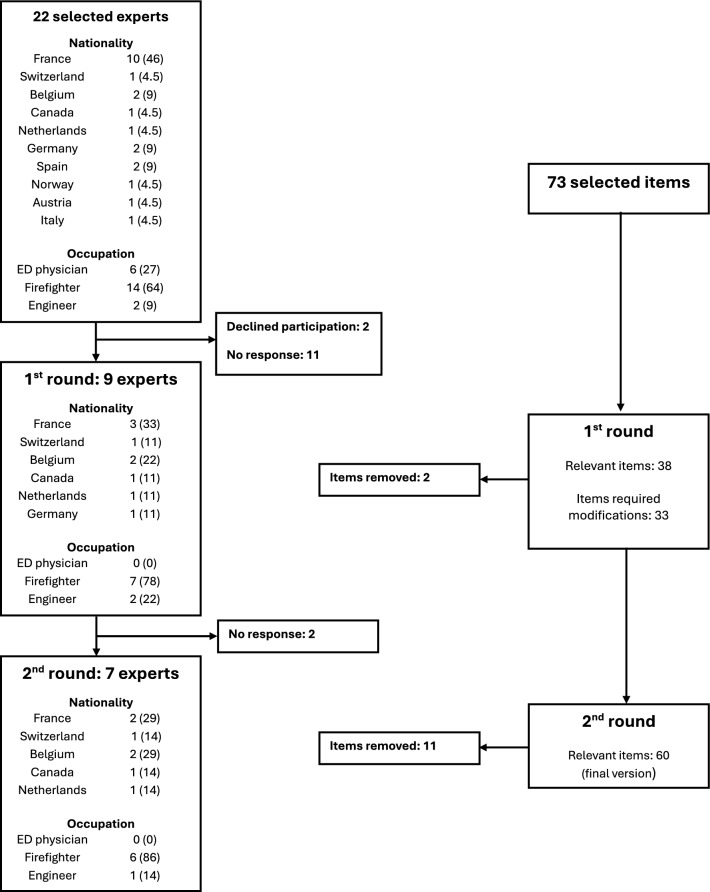
Experts’ participation and RoadRes-Q scale development. Data are presented as n (%). ED: Emergency department

In the first round, 11 experts responded to the invitation, though two declined participation, and 11 did not respond to the initial email. The first version of the scale included 73 items. After the first round, 38 items remained unchanged, 33 items required reformulation bases on comments received and 2 items were deleted (71 items). In the second round, seven experts participated. After the second round, 11 items were removed, resulting in a final version of the RoadRes-Q scale with 60 items (Table 1). Experts' participation and development of the RoadRes-Q scale are summarised in Fig. 1.

**Table 1:** Definitive roadside rescue skills scale

	Item rating		0	1	2	N.A
**Individual protection for each responder**		High-visibility vest with each responder's role marked on the back				
		Safety shoes				
		Long sleeves				
		Safety helmet				
		Blue gloves for trauma patients				
**Site security**		Approach marking by the ambulance driver				
		Positioning of the SMUR vehicle according to traffic direction (upstream if accident is in the traffic direction and downstream if in the opposite direction)				
		SMUR vehicle parked with wheels turned outward from the road axis				
		Prohibition of walking in the safety area called the “buffer zone” upstream of the accident				
		Flashing lights and hazard lights turned on				
		Facing traffic when moving on the road				
		Consideration of threatening environmental factors (electric poles, fires, etc.) and complex situations (cut cables, energy leaks, battery damage)				
		Communication of threatening environmental factors and complex situations to the rest of the SMUR team and firefighters				
		Adapting safety measures to the identified threats and complex situations without compromising the rescue of victims				
		SMUR team stays back as soon as a dangerous situation is identified				
		Engine of the crashed vehicle turned off if safety conditions allow				
		Primary stabilization performed if safety conditions and the situation allow				
		Parking brake of the crashed vehicle engaged if safety conditions allow				
**Vehicle security**		“P” position selected on the gear lever of an automatic/electric vehicle if safety conditions allow				
		Smart key kept at least 5 m away from the crashed vehicle if safety conditions allow and if easily accessible				
		No unnecessary manipulation of the crashed vehicle				
**Person rescue**	*Approach*	Searching for access points for rescuers to reach the patient				
		First visual contact made facing the patient to maintain head-neck-torso alignment				
		Head immobilization performed in the safest and most comfortable way				
		Face-to-face visual contact interrupted only when head immobilization is performed				
		Emergency evacuation (in case of cardiac arrest, respiratory arrest, uncontrolled active hemorrhage, etc.)				
		Victim prioritization				
		Prioritizing actions for care				
	*Inter-service Communication*	Clarifying the role of each responder during various actions to avoid unnecessary crowding around the victim and the vehicle				
		Consultation between emergency physician leader and the COS				
		The emergency physician leader and the COS define a medical access point				
		Immobilization technique before extraction discussed based on the planned maneuver and the patient's clinical condition				
		A rapid extraction axis is defined if an emergency evacuation is necessary				
		The improved extraction axis is defined				
		Estimated extraction time for improved extraction				
		The doctor/paramedic leader and the COS define a maximum extraction time not to be exceeded				
		Regular reassessment and communication on the situation				
		Systematic anticipation of an emergency evacuation				
		Emergency evacuation remains a possibility, and the organization of the ideal or rapid exit should not prevent this possibility				
		Situation report given to the SAMU to anticipate available and necessary hospital resources for victim care				
		Protective cover used during the cutting phase				
		Vigilance around cut areas and placement of protective shields				
		FFP2 masks for responders and victims during glass cutting				
		Helmet and safety goggles for responders near cutting areas				
	*Continuity of care*	Regularly reassess the autonomy of the oxygen bottle				
		Regularly reassess the battery of the ventilator throughout the intervention				
		Monitor always visible in the safe zone				
		Organizing equipment for good ergonomics				
		Note the time of tourniquet application in case of uncontrollable hemorrhage				
**Patient extraction**		Patient prepared for extraction (stretcher ready, vacuum mattress in place)				
		Ambulance heated in winter				
		Blanket available				
		Patient covered as soon as possible				
		Secure vascular access before patient mobilization				
		IV lines clamped before patient mobilization				
		Agreement of all actors involved in patient mobilization obtained before extracting the patient				
		Head-neck-torso alignment preserved				
		Intubation tube secured by the emergency physician during transfer				
		Remove unnecessary medical equipment and ensure that the rest of the equipment follows the patient during extraction				
		Check vascular access patency after vehicle extraction				

N.B: Grayed items should be performed if the mobile emergency and resuscitation unit (SMUR) team arrives first. COS: Rescue Operations Commander; SAMU: Emergency Medical Communication Center; FFP2: Filtering Facepiece Particles masks filtering at least 94% of aerosols with an average size ranging from 0.06 to 0.45 µm; IV: Intra-venous

### Psychometric testing: validity and reliability analysis

The RoadRes-Q scale had excellent internal consistency with no redundancy between items, as assessed by a Cronbach’s alpha coefficient of 0.86.

Concerning reliability, several statistical analyses were performed. The T-test comparison of group means showed no significant difference between observers (*p* = 0.10). Accordingly, the F-test comparison of variances between observers showed no significance (*p* = 0.21).

The ICC calculation showed a weak concordance between 2 observers, with an ICC of 0.48. The R^2^ coefficient using linear regression showed a mediocre level of reliability between observers, with a value of 0.47.

### Simulation sessions evaluation

Demographic data, satisfaction, knowledge, TAPAS and CTS could only be assessed on the first day of the simulation due to technical issues. A third of participants were under 35 years old, and half had less than 5 years’ experience in the ED. Most participants (89%) had previous roadside rescue training, and half had completed more than 20 interventions. Participant demographics are summarised in Table 2.

**Table 2:** Participants characteristics in simulation sessions (J1)

Demographic criteria	
Male sex	5 (83)
Age (years)	
*25–30*	1 (17)
*30–35*	1 (17)
*35–40*	2 (33)
> *40*	2 (33)
Length of experience at the SAMU (years)	
*1–5*	3 (50)
*5–10*	1 (17)
> *10*	2 (33)
Previous roadside rescue interventions performed	
1*–10*	2 (33)
*10–20*	1 (17)
> *20*	3 (50)
Previously attended a simulation session	
*Yes, in Poitiers*	3 (50)
*Yes, in another center*	2 (33)
*No*	1 (17)
Previously attended a roadside rescue training program	
*Yes, in Poitiers*	3 (50)
*Yes, in another center*	2 (33)
*No*	1 (17)

Data are n (%); SAMU: emergency medical communication centre

Satisfaction was high among participants, with all respondents rating improvements in inter-team relations as 5/5 and recommending the training to their colleagues. Overall satisfaction scores ranged from 8 to 10 out of 10, with an average of 9 across all participants. All the results about the satisfaction survey are described in the Table 3.

**Table 3:**
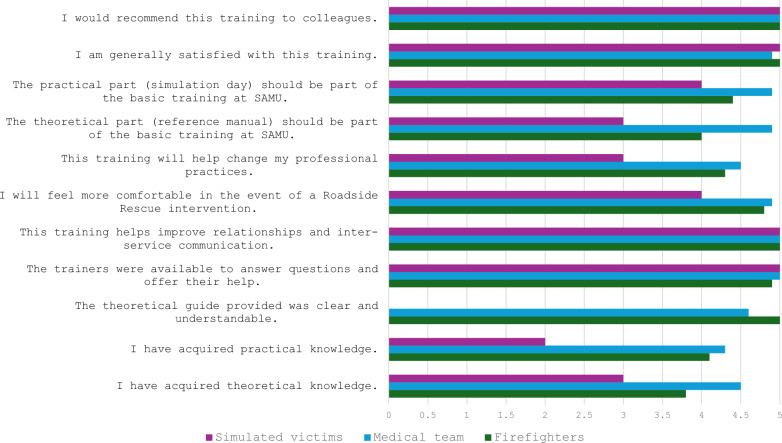
Satisfaction assessment for participants in simulation sessions (J1)

SAMU: emergency medical communication centre

Knowledge improved after simulation, as reflected by differences between pre-test and post-test scores (4.2 ± 1.1 versus 12.0 ± 2.0, difference: 7.8 [IC 95%, 4.6–11.0], *p* = 0.0025).

The average TAPAS score for all observers across all scenarios was 67/100 ± 15. Similarly, the average CTS score was 97/130 ± 14.

## Discussion

### Main results

In this study, which involved nine international experts in a Delphi method, a 60-item scale was developed to assess the roadside rescue performance of medical teams. Subsequently, this evaluation scale was used during six roadside rescue scenarios, involving 12 healthcare professionals and 32 firefighters. This allowed for the validation process to be conducted, demonstrating very high validity and good reliability. Additionally, the evaluation of the simulation-based training process for roadside patient care showed high learner satisfaction, with improvements in knowledge and the acquisition of both technical and non-technical skills. To our knowledge, this is the first study addressing this topic by creating an evaluation tool and testing its psychometric properties during simulation sessions.

### The RoadRes-Q scale development and its psychometric properties

Using a Delphi method involving nine international experts, we created a 60-item skills RoadRes-Q scale that covers all essential techniques and procedures for caring patients during roadside rescues. The scale was developed to comprehensively evaluate the critical competencies required in these high-stakes scenarios, ensuring both clinical and operational efficacy. The internal validity of the RoadRes-Q scale is supported by a Cronbach's alpha coefficient of 0.86, demonstrating excellent internal consistency and confirming that the items are non-redundant. However, the results related to inter-observer reproducibility were mixed. While comparison of mean scores across observers showed no significant differences, the ICC indicated weak agreement between observers. It would be interesting to explore whether certain types of observers, such as physicians versus firefighters, experienced greater difficulty in agreeing on specific criteria. This discrepancy may be attributed to the subjective nature of non-technical skills, such as leadership, teamwork, and communication, which are often more challenging to assess consistently across different observers [7].

The simulation-based training provided a realistic platform to apply the RoadRes-Q scale. Ensuring the quality of training is critical for the validation of the assessment scale, as it guarantees that the scenarios and learning conditions reflect real-world situations as accurately as possible. A well-structured, high-quality simulation provides a reliable platform for observers to apply the scale in a meaningful way. If the training itself is of poor quality, it could introduce bias into the assessment of team performance and obscure the true effectiveness of the scale. Therefore, the thorough assessment of both knowledge improvement and satisfaction ensures that the context in which the scale is tested is rigorous and conducive to the collection of valid and reliable data. The satisfaction levels, significant knowledge improvement, clinical skills and teamwork performances seen among participants reinforce the RoadRes-Q scale effectiveness in contributing to common operational culture among roadside rescue actors.

This present study aligns with previous research in emergency medicine that highlights the importance of robust evaluation scales. While the scientific literature is rich in studies focusing on creation of scores or validation of measurement scales in emergency settings [23, 30–36], assessment of roadside rescue skills remains relatively unexplored. In 2022, Nutbeam et al. developed a 91-item scale for best practices in handling entrapped victims using a Delphi method [37]. Although comprehensive, this scale lacks the structured, time-sensitive elements needed for use in simulation training, as it does not provide a mechanism for evaluating the real-time performance of rescue teams. The RoadRes-Q scale fills this gap by offering a structured evaluation method designed for use during simulations, allowing for real-time performance evaluation and feedback.

### Limitations and strengths

This study has some limitations. Firstly, the response rate from experts involved in the Delphi process was relatively low, with only half of them responding to the initial solicitation. Additionally, the relatively low number of physicians involved in the development of the scale may have impacted its comprehensiveness, particularly regarding medical management. Despite this, the high internal consistency of the scale suggests that items included are non-redundant and effectively cover all the necessary skills. While the participating experts provided valuable input, a higher participation rate would have increased the diversity of perspectives and potentially strengthened the consensus. To improve expert engagement in future studies, it may be beneficial to implement regular reminders and possibly incentives to encourage broader participation. Secondly, although this is not an objective of the present study, the generalisation of results to other contexts could be questioned. The RoadRes-Q scale was assessed for use in the French prehospital care system, which follows a “stay and play” model of patient management. This model contrasts with the “scoop and run” approach used in many Anglo-Saxon countries, where the focus is on rapid transport to the hospital [4]. However, the RoadRes-Q scale was developed with the help of international experts, some of whom use paramedics and the “scoop and run” approach and we believe that this scale could also be suitable for this model. The scale focuses specifically on roadside rescue skills rather than broader medical competencies, making it adaptable to both French pre-hospital teams operating under the “stay and play” model and paramedics practicing the “scoop and run” approach. Finally, inter-observer agreement was weak, which could compromise the objectivity of the assessment. This can be attributed to the limited number of simulation scenarios used for validation, and the lack of dedicated training for observers in applying the scale.

This study has also several strengths. Firstly, the international multidisciplinary approach to developing the reference manual and the RoadRes-Q scale ensured input from a diverse range of experts, fostering a shared culture among roadside rescue stakeholders. This collaborative process is crucial in creating a common framework that aligns with the principle of “training together those who work together” through multi-professional simulation exercises. Secondly, by providing a standardised scale, it allows trainers to objectively assess both technical and non-technical performance, identify areas for improvement, and ultimately enhance the quality of simulation-based training. The high levels of participant satisfaction and the improvement in knowledge following the training suggest that the scale has great potential in fostering a shared operational culture between medical teams and firefighters, thereby improving collaboration and coordination in real-world rescue scenarios. Finally, although the RoadRes-Q scale was developed with input from expert firefighters and healthcare professionals, its applicability extends beyond this group. The simulation-based validation included professionals with varying levels of experience, demonstrating that the scale can be used both as an assessment tool and as a structured training framework to enhance the skills of less experienced health care professionals.

### Use of RoadRes-Q scale and future research

By providing a structured method for performance assessment, the RoadRes-Q scale seeks to enhance the quality of training, foster a common operational culture, and ultimately improve patient outcomes during real-life emergencies. The RoadRes-Q scale was developed to address the significant gap in current training frameworks, where no standardised method exists to evaluate skills of emergency teams during high-pressure interventions.

Looking ahead, future research could focus on refining the scale to improve inter-observer reliability, particularly in the assessment of non-technical skills. To address this issue, several strategies could be implemented in future applications of the scale. First, standardised training for observers could significantly improve inter-rater agreement. Structured sessions focusing on the use of the RoadRes-Q scale, alongside calibration exercises involving real or recorded simulations, would help align evaluation criteria and reduce subjective variability. Additionally, video recordings of simulation sessions could be used for retrospective evaluation, allowing observers to review performances collectively and discuss discrepancies in scoring. This method, widely used in simulation research, has proven effective in enhancing inter-observer reliability by enabling shared reflection and calibration of assessments. Implementing these strategies could improve the consistency and objectivity of evaluations in future iterations of the scale [30, 31]. Additionally, further studies could explore the long-term impact of using this scale on actual rescue performance and patient outcome in real-world settings. Expanding the use of this scale across various geographical and cultural contexts would provide valuable insights into its broader applicability and effectiveness. Additional studies are needed to evaluate the applicability of this scale in different prehospital care systems, such as those found in the UK, the US, or other countries with varied emergency response practices.

## Conclusion

The creation and validation of the RoadRes-Q scale mark a significant step forward in the training and evaluation of multidisciplinary emergency teams involved in roadside rescue operations. With strong internal consistency and high participant satisfaction, this scale offers a structured, objective method for assessing both technical and non-technical skills under high-pressure conditions. While improvements in inter-observer reliability are needed, particularly through enhanced evaluator training and the use of video-assisted assessments, the RoadRes-Q scale has the potential to become a vital tool in fostering a shared operational culture between medical teams—or paramedics—and firefighters. By standardising performance evaluation, it not only enhances the quality of simulation-based training but also holds the promise of improving real-world patient outcomes during road traffic accidents. Future research should focus on refining the scale reproducibility and exploring its broader applicability across diverse emergency response systems.

## Data Availability

The datasets generated and analysed during the current study are available from the corresponding author upon reasonable request. All relevant data have been anonymised to protect participant confidentiality. The assessment scale and related materials used in the study can also be made available for educational or research purposes, subject to approval by the research team.

## Abbreviations

AAR: After action review
CIC: Clinical investigation center
CHU: Centre Hospitalier Universitaire (University Hospital)
COS: Commandant des Opérations de Secours (rescue operations commander)
ICC: Intraclass correlation coefficient
INSERM: Institut National de la Santé et de la Recherche Médicale (National Institute of Health and Medical Research)
KSA: Knowledge, skills and attitudes
MCQ: Multiple choice questionnaire
SAMU: Service d'Aide Médicale Urgente (emergency medical assistance service)
SDIS: Service Départemental d'Incendie et de Secours (Departmental Fire and Rescue Service)
SMUR: Service Mobile d'Urgence et de Réanimation (mobile emergency and resuscitation service)
TAPAS: Team average performance assessment scale
