# Correction: Creation and validation of a roadside rescue skills scale for training pre-hospital medical teams: the RoadRes-Q scale

**DOI:** 10.1186/s13049-025-01389-0

**Published:** 2025-05-01

**Authors:** Killien Lavabre, Nicolas Marjanovic, Denis Oriot, Mathilde Chenu, Adrien Gransagne, Michel Gentilleau, Anthony Moreau, Paul Contal, Olivier Mimoz, Bertrand Drugeon, Road Rescue Study Group, Jean Gabriel Clouet, Tom Van Esbroeck, Sébastien Guay, Ruediger Knoll, Julien Manesse, Frans Van Maurik, Bart Noyens, Raphael Mouth, Cédric Rigollet

**Affiliations:** 1 Service Des Urgences Adultes Et SAS 86/SMUR, CHU de Poitiers, 2 Rue de La Milétrie, Poitiers Cedex, 86021 France; 2 INSERM CIC-1402, IS- ALIVE, Université de Poitiers, Poitiers, France; 3 ABS Lab, Faculté de Médecine Et de Pharmacie, Université de Poitiers, Poitiers, France; 4 Service Des Urgences Et SAMU, Hôpital Saint Louis, La Rochelle, France; 5 Service Départemental d’Incendie Et de Secours 86, Chasseneuil du Poitou, France; 6 PHAR2 - INSERM U1070, Université de Poitiers, Poitiers, France

**Correction: Scand J Trauma Resusc Emerg Med 33, 56 (2025)**

**https://doi.org/10.1186/s13049-025–01370-x**

After the original article [1] was published, the authors reported that all given and family names had been mistakenly reversed. The corrected names provided in this Correction are accurate.

The original article has been corrected.
